# The glucose uptake inhibitor SgrS is induced by D-serine yet does not contribute to growth arrest in enterohaemorrhagic Escherichia coli

**DOI:** 10.1099/mic.0.001648

**Published:** 2026-01-09

**Authors:** Ella Rellis, Nicky O'Boyle

**Affiliations:** 1 Department of Microbiology, School of Genetics & Microbiology, Moyne Institute of Preventive Medicine, Trinity College Dublin, Dublin, D02 A2H0, Ireland

**Keywords:** *E. coli*, metabolism, regulation, small RNA, stress response, transporter

## Abstract

Bacterial pathogens sense and respond to host-derived metabolites to regulate virulence and establish successful infections. d-Serine, an amino acid abundant in some extraintestinal environments but scarce in the intestine, functions as a key niche-specific signal that influences the tissue tropism of different *Escherichia coli* pathotypes. In enterohaemorrhagic *E. coli*, a major foodborne pathogen causing severe human disease, d-serine exposure triggers significant transcriptional changes distinct from those seen in extraintestinal pathotypes. Recent publication of our extensive d-serine pathotype transcriptome data on the open database MORF included genome reannotation. This revealed the previously unannotated small regulatory RNA SgrS as being the most significantly upregulated transcript in response to d-serine exposure. Despite its canonical role in managing glucose-phosphate stress by inhibiting glucose uptake, here, we show that d-serine-mediated growth inhibition occurs independently of SgrS and extends beyond glucose metabolism, affecting growth across diverse carbon sources with varying transport mechanisms. Growth inhibition persisted following the deletion of *sgrS* and could not be alleviated by pyruvate or pantothenate supplementation, while l-serine provided partial rescue, suggesting disruption of fundamental metabolic processes. The novel observation that d-serine induces SgrS suggests a wider regulatory role beyond managing glucose phosphate stress during impeded glycolysis. Moreover, we describe key distinctions between d-serine toxicity in EHEC and previous reports with laboratory *E. coli* strains, highlighting the importance of strain-specific metabolic and regulatory architecture in response to host-associated cues.

## Data Availability

Raw RNA-Seq read data associated with this manuscript is available under ENA accession number PRJEB36547. The sequence of the empty pSS (XylS/Pm) plasmid has been deposited at NCBI under accession number PX119074.

## Introduction

*Escherichia coli* is one of the most extensively studied bacterial species and exemplifies microbial adaptability, ranging from harmless commensals to highly virulent pathogens. Colonization begins shortly after birth, with *E. coli* establishing itself as one of the earliest and most abundant residents of the human gastrointestinal tract [1]. Through horizontal gene transfer and the acquisition of mobile genetic elements, certain *E. coli* lineages have evolved into distinct pathotypes capable of causing intestinal and extraintestinal infections. Among these pathotypes, enterohaemorrhagic *E. coli* (EHEC), particularly serotype O157:H7, stands as one of the most clinically significant foodborne pathogens worldwide. Unlike many bacterial pathogens, EHEC has an exceptionally low infectious dose of ~100 cells, facilitating rapid transmission and considerable epidemic potential [2]. The global burden of EHEC infections is estimated at 2.8 million cases annually, with particularly high prevalence in regions with intensive cattle farming [3].

EHEC is a zoonotic pathogen, asymptomatically colonizing the terminal rectum of ruminants and disseminated through faecal shedding. Human infection occurs predominantly through the faecal-oral route, typically through ingestion of contaminated food or water [4]. Clinical presentation ranges from abdominal cramps and haemorrhagic colitis to haemolytic uraemic syndrome (HUS) in ~10% of cases, particularly in young children and the elderly. HUS, characterized by haemolytic anaemia, thrombocytopenia and renal failure, has a 3–5% mortality rate, with long-term complications in survivors [3].

The pathogenicity of EHEC is driven primarily by two major virulence factors encoded on mobile genetic elements. The locus of enterocyte effacement (LEE) encodes a type III secretion system (T3SS) that manipulates host cell signalling, disrupts tight junctions and induces the formation of attaching and effacing lesions on the intestinal mucosa [5]. This intimate attachment distinguishes EHEC from commensal *E. coli*, which remain in the intestinal lumen. In parallel, Shiga toxins (Stx1 and/or Stx2), encoded by lambdoid prophages, inhibit host protein synthesis by cleaving 28S rRNA [6]. Shiga toxins target renal endothelial cells through Gb3 receptors, contributing to the acute renal failure seen in HUS [7]. The presence of both the LEE-encoded T3SS and Shiga toxins forms a distinct virulence profile, distinguishing EHEC from other enteric pathogens and contributing to its clinical severity [8].

Current treatment is limited to supportive therapy, as conventional antibiotics risk exacerbating disease through prophage induction and increased Stx production [9]. EHEC virulence is tightly regulated by its ability to sense environmental signals. Understanding these regulatory pathways presents a potential avenue for novel therapeutic strategies that suppress virulence without inducing toxin production, while also minimizing selective pressure for the emergence of antibiotic resistance. Among these signals, host-derived metabolites play a critical role in modulating EHEC virulence. One such metabolite is d-serine, a non-canonical amino acid abundant in some extraintestinal niches, produced by serine racemase in the brain, where it functions in neurotransmission [10]. Its concentrations vary widely across the body, exceeding levels of 1 mM in the urinary tract and central nervous system while remaining virtually absent (~1 µM) in the intestines, potentially shaping the niche distribution of *E. coli* pathotypes [11].

In uropathogenic *E. coli*, the *dsdCXA* operon enables d-serine detoxification, allowing colonization of d-serine-rich environments, such as the urinary tract. This operon encodes a transcriptional regulator (DsdC), a transporter (DsdX) and a deaminase that converts d-serine into pyruvate and ammonia (DsdA). In contrast, EHEC lacks a functional *dsdCXA* operon, characterized by the absence of *dsdC*, a truncated *dsdX* and the presence of *dsdA*. Despite the absence of a functional DsdX, EHEC imports d-serine through alternative transporters CycA and SstT, leading to cytosolic accumulation and toxicity through mechanisms that are not yet fully understood [11]. Additionally, d-serine can be misincorporated into peptidoglycan precursors, competing with d-alanine, disrupting cell wall synthesis and potentially exacerbating toxicity [12].

Beyond direct toxicity, d-serine downregulates the LEE-encoded T3SS, preventing EHEC from colonizing unfavourable niches where d-serine is abundant. The rarity of bacteria carrying both the LEE T3SS and an intact *dsdCXA* locus suggests strong evolutionary pressure against attaching and effacing pathogens colonizing such environments [8]. Exposure to d-serine also induces an SOS-like response in EHEC by activating the SOS antirepressor RecA and increasing mutation rates, likely an adaptive mechanism to mitigate d-serine stress through accelerated evolution [13].

While d-serine plays a key role in EHEC niche restriction and virulence regulation, the molecular mechanisms by which EHEC responds to and potentially mitigates d-serine stress remain to be understood. In this study, we expand on a previous transcriptomic analysis of EHEC under d-serine stress [14]. This study revealed significant global changes in gene expression, which we now reveal also includes a small regulatory RNA encoding gene, *sgrS*, as the most significantly upregulated transcript. This upregulation was unexpected given the canonical role of SgrS in managing glucose-phosphate stress, which occurs when phosphosugars such as glucose-6-phosphate or its non-metabolizable analogues accumulate to toxic levels, disrupting carbon metabolism and inhibiting growth [15]. Although the exact molecular cause of this stress is yet to be completely characterized, the regulatory response mechanisms are well established.

In most *Enterobacteriaceae*, this stress is regulated by the SgrST system, consisting of SgrS, a small regulatory RNA, and SgrT, a small protein encoded within the 5′ region of SgrS. Upon activation by SgrR, SgrS base pairs with mRNAs encoding the phosphoenolpyruvate-dependent phosphotransferase system (PTS) transporters, *ptsG* (EIICB^Glc^) and *manXYZ* (EIIABCD^Man^), leading to translational repression and degradation via the RNA degradosome [16]. While SgrST is generally conserved across *Enterobacteriaceae*, significant variations exist among strains. In *E. coli* O157:H7, a point mutation in the 5′ region of SgrS changes the *sgrT* start codon from ATG to ATT, preventing SgrT expression and making SgrS the sole active component of the system [17]. Complementation studies suggest that, despite lacking SgrT, the regulatory capacity of SgrS likely remains functionally relevant and physiologically significant in responding to metabolic stress via post-transcriptional regulation of *ptsG* and *manXYZ* mRNAs [18].

d-Serine severely inhibits growth in glucose-containing minimal media, highlighting its role as a niche-restricting metabolite in the host environment. Although SgrS is strongly upregulated under d-serine stress, its contribution to growth arrest, toxicity mitigation and metabolic stress adaptation remains unclear. This study investigates the functional role of SgrS in mediating EHEC’s response to d-serine and elucidates broader mechanisms underlying d-serine toxicity by assessing its impact on metabolic pathways beyond glucose utilization and evaluating the requirement of SgrS for effective stress adaptation. While minimal media do not fully replicate *in vivo* conditions, they provide a controlled system for dissecting stress responses and regulatory interactions. Finally, this study assesses the link between d-serine exposure, SgrS activation and virulence gene regulation to comprehensively explore the role of SgrS in the regulation of traits previously described as being altered by d-serine.

## Methods

### Bacterial culture conditions

Single bacterial colonies were routinely inoculated in Miller’s formulation lysogeny broth (LB) and cultured at 37 °C with shaking at 200 r.p.m. Where appropriate, LB and LB agar were supplemented with kanamycin (50 μg ml^−1^) (Merk), ampicillin (100 μg ml^−1^) (Merk), chloramphenicol (25 μg ml^−1^) (Carl Roth) or hygromycin B (100 μg ml^−1^) (Carl Roth). M9 minimal media was prepared using 5X M9 salts supplemented with CaCl_2_, MgSO_4_ and specified carbon sources. For assays involving d-serine (Carl Roth), a final concentration of 1 mM was added, consistent with physiologically relevant levels. For assays requiring T3SS induction, overnight LB cultures were diluted 1 : 100 in MEM-HEPES (Merk). Bacterial stocks were stored at −72 °C and cryopreserved in LB supplemented with 20% (v/v) glycerol. Bacterial strains used in this study are provided in Table S1 (available in the online Supplementary Material).

### Growth assays

Bacterial growth assays were conducted in 50 ml Erlenmeyer flasks (Thermo Fisher Scientific) containing 10 ml M9 minimal medium with a single carbon source at a final concentration of 0.4% (w/v), unless otherwise stated. Carbon sources included glucose (Thermo Fisher Scientific), mannose (TCI), arabinose (Merk), galactose (TCI), glycerol (Thermo Fisher Scientific), sucrose (SLS), lactose (SLS) or maltose (TCI). l-serine (Carl Roth) and calcium pantothenate (Thermo Fisher Scientific) were added at final concentrations of 1 mM and 1 µg ml^−1^, respectively. For assays involving pyruvate, sodium pyruvate (Thermo Fisher Scientific) was used at a final concentration of 36 mM, either added with glucose or substituted for the standard carbon source. Media were inoculated with overnight LB cultures diluted 1 : 100 and incubated at 37 °C with shaking at 200 r.p.m. All growth assays were performed both with and without d-serine supplementation. OD was measured at 600 nm (OD_600_) every hour using an Eppendorf BioPhotometer Model (#6131). All assays were performed in biological triplicate.

### Specific growth rate

Specific growth rate (SGR) was calculated as *µ* = Δln OD_600_ nm / Δ*t*, where ‘Δln OD_600_’ nm is the change in the natural logarithm of the OD_600_ nm and ‘*t*’ is time in hours. SGR values were derived from OD_600_ measurements taken during the exponential phase based on triplicate assays.

### Generation of *sgrS* deletion using Lambda Red recombination

A deletion of *sgrS* from EHEC TUV93-0 was generated using the Lambda Red recombineering system. A four-fragment construct was assembled using linearized pACYC184 (primers pACYC-lin-F and pACYC-lin-R), the kanamycin cassette from pKD4 (primers pKD-F and pKD-R) and ~600 bp homology sequences flanking the *sgrS* locus (primers SgrS-DelA and SgrS-DelB, SgrS-DelC and SgrS-DelD). Fragments were designed with 25 bp overhang sequences to facilitate *in vitro* assembly using NEBuilder HiFi DNA Assembly Master Mix (New England Biolabs) (50 °C, 1 h). The assembled construct was transformed into DH5*α* via heat shock (42 °C, 45 s), followed by recovery in super optimal complete (SOC) medium (37 °C, 200 r.p.m., 2 h) and selection on LB-kanamycin agar. Successfully assembled deletion plasmids were identified by colony PCR, purified by miniprep and confirmed by Sanger sequencing. Deletion constructs were amplified from >1,000-fold diluted sequence-verified plasmid DNA using pACYC-seq2-F and pACYC-check-R primers before being column purified to serve as the Lambda Red template. TUV pSIM18 cells were incubated at 28 °C to early log phase (OD_600_ of ~0.4) and induced at 42 °C for 15 min. Cells were harvested, washed and electroporated (2.5 kV) with 1 µg of Red DNA. Cells were recovered in SOC medium (37 °C, 200 r.p.m., 2 h) and recombinants were selected for on LB-kanamycin agar. Positive mutants were verified by colony PCR, antibiotic spot-plating to ensure loss of pSIM18 and Sanger sequencing. Plasmid and PCR-amplified DNA were extracted using a QIAprep Miniprep Kit or PCR Purification Kit (Qiagen), with DNA eluted in nuclease-free water, and quantified using a NanoDrop spectrophotometer (DeNovix). All plasmids and primer sequences used in this study are listed in Tables S2 and S3, respectively.

### Complementation of *sgrS* using a synthetic XylS/Pm expression plasmid

A construct (pSS-XylS/Pm, hereby referred to as pSS) comprising the benzoic acid inducible Pm promoter and the transcriptional regulator encoding gene *xylS* from pSEVA238 [19] together with ColE1 origin of replication, beta lactamase and low copy number enhancer gene *rop* from pBR322 [20] was synthesized by Twist Bioscience. The sequence of this construct has been uploaded to NCBI (accession: PX119074). The associated plasmid map generated from this sequence is shown in the supplementary information (Fig. S1). This vector was linearized 80 bp downstream of the Pm promoter by amplification with Q5 high-fidelity polymerase (New England Biolabs) using primers pSS-lin-F and pSS-lin-R before gel extracting and purifying using a QIAprep Miniprep Kit (Qiagen). A region spanning the transcriptional start site to 68 bp downstream of the 3′ end *sgrS* was also amplified using primers sgrS-pSS-F and sgrS-pSS-R. The resulting fragment was assembled downstream of the Pm promoter using NEB HiFi DNA Assembly Master Mix (New England Biolabs) and transformed into *E. coli* DH5*⍺* before selecting on LB-ampicillin plates. Correct assembly was confirmed by colony PCR using primers pSS-check-F and pSS-check-R2 and Sanger sequencing of plasmid miniprep DNA derived from clones displaying appropriate banding. The plasmid was then transformed into the *sgrS* deletion mutant by electroporation followed by selection on ampicillin. To verify appropriate deletion and complementation, the WT, *sgrS* deletion mutant and complemented mutant carrying pSS-*sgrS* were plated on LB, LB with 0.5% *⍺*-methylglucoside (*⍺*-MG) and LB with 0.5% *⍺*-MG and 1 mM 3-methylbenzoic acid before incubating at 37 °C overnight and imaging.

### Fluorescence-based reporter assays

TUV93-0 WT and Δ*sgrS* strains carrying the plasmids pDUAL or pLEE1/recA were used to assess the promoter activity of *LEE1p* and *recAp*. Overnight LB cultures were diluted 1 : 100 in 5 ml of MEM-HEPES with and without 1 mM d-serine and incubated at 37 °C, 200 r.p.m. A 200 µl aliquot of each culture was transferred to a flat-bottom 96-well microtitre plate for measurement. OD_600_ and fluorescence intensity measurements (GFP: Ex485/Em528 nm; RFP: Ex590/Em635 nm) were read using a BioTek Synergy HTX Reader. Background fluorescence was determined using strains carrying the promoterless pDUAL plasmid. Fluorescence data was background-corrected by subtracting the fluorescence intensity of the strain carrying pDUAL for each time point. Corrected fluorescence intensities were normalized relative to OD_600_ nm, expressed as relative fluorescence units (RFU). All assays were performed in triplicate.

### Statistical analysis

Statistical significance was determined by Welch’s t-test using GraphPad Prism 10.5.0.

## Results

**Fig. 1.**
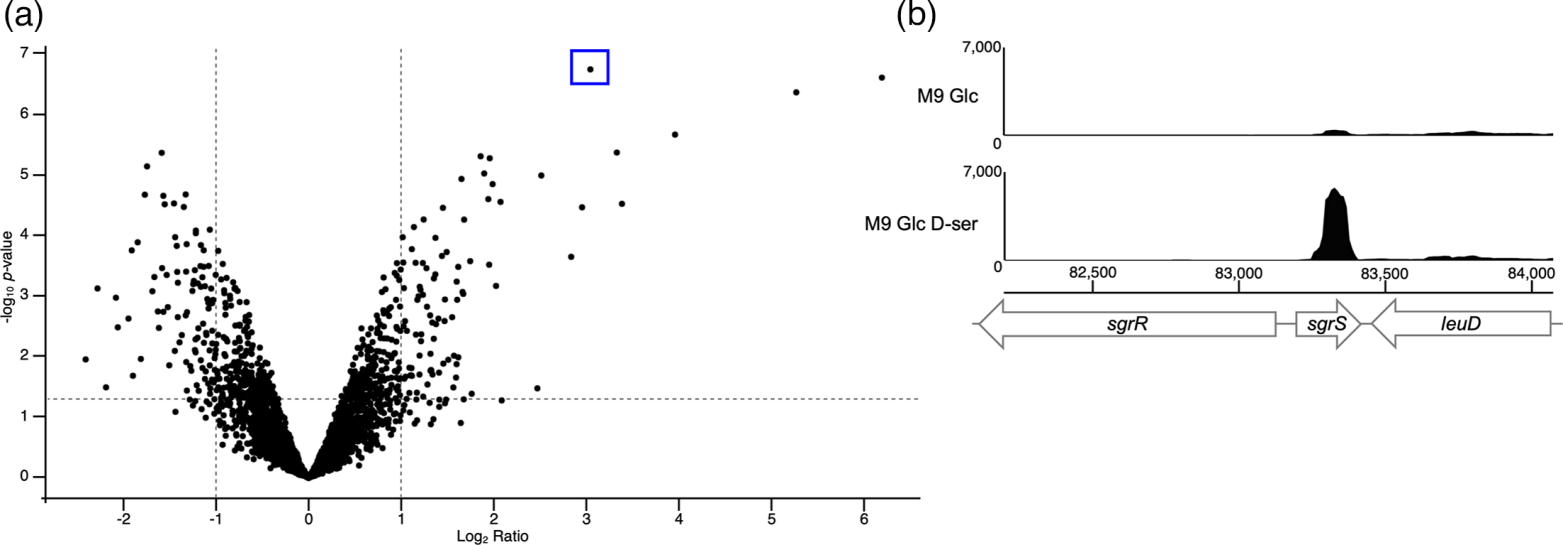
*sgrS* is the most significantly upregulated transcript in response to d-serine in EHEC. RNA-Seq data showing the transcriptomic response of EHEC to d-serine. (**a**) Volcano plot indicating that SgrS (blue box) is upregulated 8.26-fold (adjusted *P*=0.0004). Figure adapted from MORF database [14,21]. (**b**) Track maps showing RNA read density in the *sgrS* locus for representative samples ERR3861817 (M9 Glc) and ERR3861819 (M9 Glc d-ser). Samples were chosen on the basis of having the most similar total reads mapped count (7,957,661 and 7,218,584 reads mapped, respectively).

To explore how different *E*. coli pathotypes respond to d-serine at the transcriptomic level, we previously analyzed RNA-Seq data from EHEC, uropathogenic *E. coli* (UPEC) and neonatal meningitis-associated *E. coli* strains exposed to d-serine [14]. Comparative analysis revealed distinct gene expression profiles, reflecting each pathotype’s niche-specific adaptations. Our dataset was recently uploaded to the MORF repository [21], a process which involved genome reannotation to allow for direct orthologue comparison. This revealed the previously excluded sRNA encoding gene *sgrS* as the most significantly upregulated gene in EHEC under d-serine exposure (Fig. 1). Given that growth of EHEC in M9 with glucose as a sole carbon source is strongly inhibited by d-serine [14] and that the canonical role of SgrS is inhibition of glucose uptake, this finding suggested a previously unrecognized mechanism for d-serine-induced growth arrest.

While the deletion in *sgrS* was under construction, its role was first tested indirectly. To determine whether d-serine-mediated growth arrest was specifically linked to SgrS-dependent inhibition of the PTS-dependent glucose transporters PtsG and ManXYZ, growth was assessed in M9 minimal medium supplemented with various sole carbon sources. Both PTS-transported and non-PTS-transported sugars were tested to determine whether d-serine inhibition extends beyond PTS-dependent pathways, particularly those regulated by SgrS (Table 1).

**Table 1.** Sugar transport systems supporting carbon metabolism in *E. coli*

Sugar	Transporter(s)	PTS	ABC	Facilitated diffusion	Proton symport	SgrS	Reference
Glucose	PtsG; ManXYZ; NagE; MglBAC; GalP	**^✔^**	**^✔^**		**^✔^**	**◆**	[29]
Mannose	ManXYZ	**^✔^**		**^✔^**		**◆**	[29]
Galactose	GalP; MglBAC		**^✔^**		**^✔^**		[29]
Arabinose	AraE; AraFGH		**^✔^**		**^✔^**		[29]
Glycerol	GlpF			**^✔^**			[29]
Lactose	LacY				**^✔^**		[30]
Sucrose	CscB; ScrA	**^✔^**			**^✔^**		[29]
Maltose	MalEFGK		**^✔^**				[29]

✔ indicates transporter families associated with each sugar (phosphoenolpyruvate-dependent phosphotransferase system is abbreviated to PTS, and ATP-binding cassette transporter is abbreviated to ABC); **◆** denotes known regulatory interactions between sugar transporter and SgrS.

**Fig. 2.**
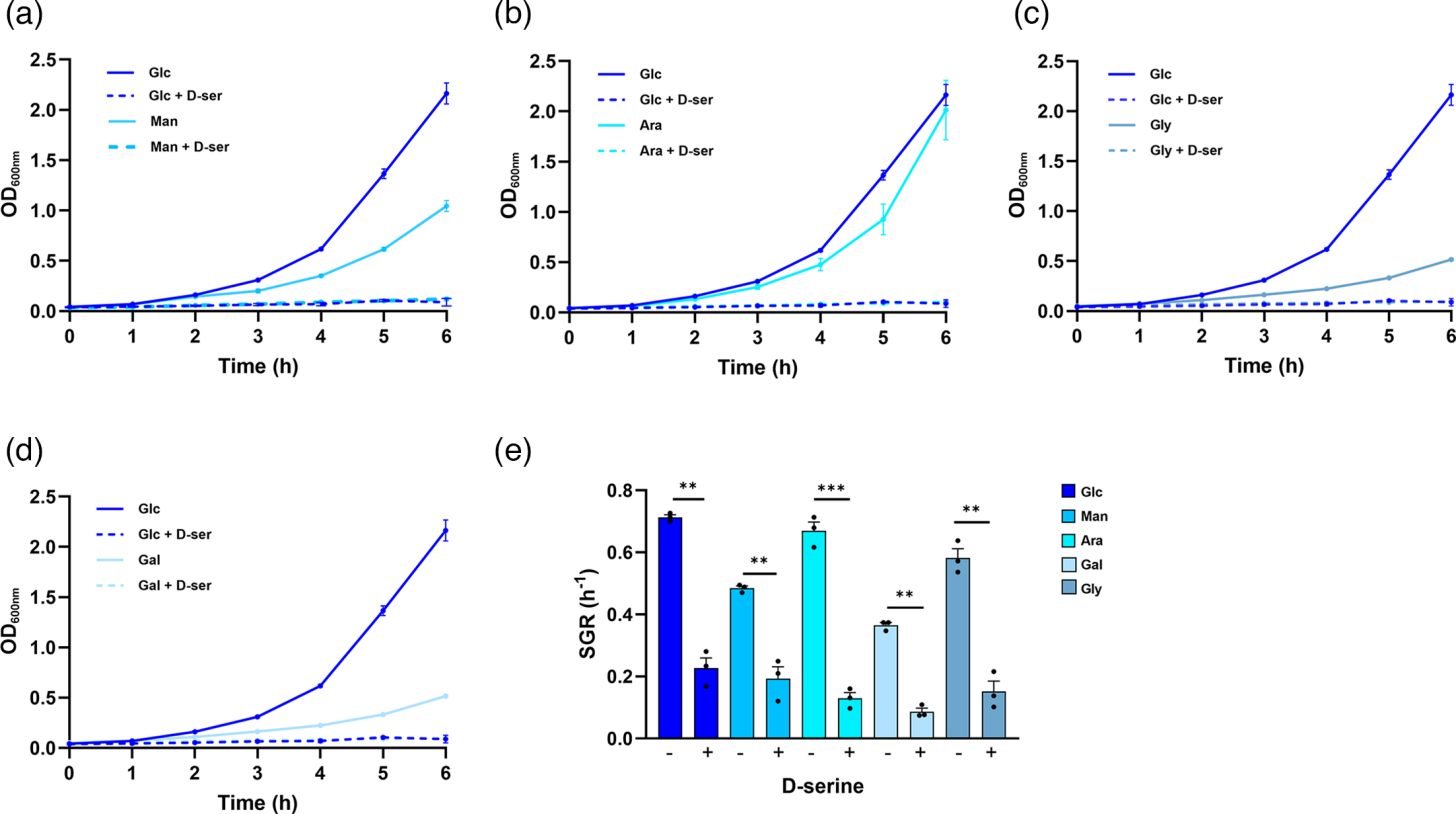
d-Serine inhibits TUV93-0 growth in M9 with diverse monosaccharide sole carbon sources. (**a–d**) Growth curves of TUV93-0 in M9 with either glucose or an alternative monosaccharide each at 0.4% (w/v), with (dashed lines) and without (solid lines) 1 mM d-serine. Error bars indicate the sem. (**e**) Exponential growth rates for each monosaccharide. Black dots indicate individual replicates and error bars show sem. Statistical significance: *P*<0.05 (*), *P*<0.01 (**), *P*<0.001 (***) and *P*>0.05 (ns). The data is representative of three biological replicates.

Growth was assessed in M9 minimal medium using glucose as a reference, alongside mannose, arabinose, galactose and glycerol as sole carbon sources, each of which acts as a substrate for different transport systems and transporter families. The different carbon sources supported varying growth rates under control conditions, reflecting differences in uptake and metabolism. d-Serine strongly inhibited growth across all monosaccharides tested (Fig. 2a–d). Exponential growth rate analysis confirmed a significant reduction in growth in all conditions (*P*<0.05) (Fig. 2e), suggesting that d-serine-mediated growth inhibition is not restricted to specific sugar transport mechanisms.

**Fig. 3.**
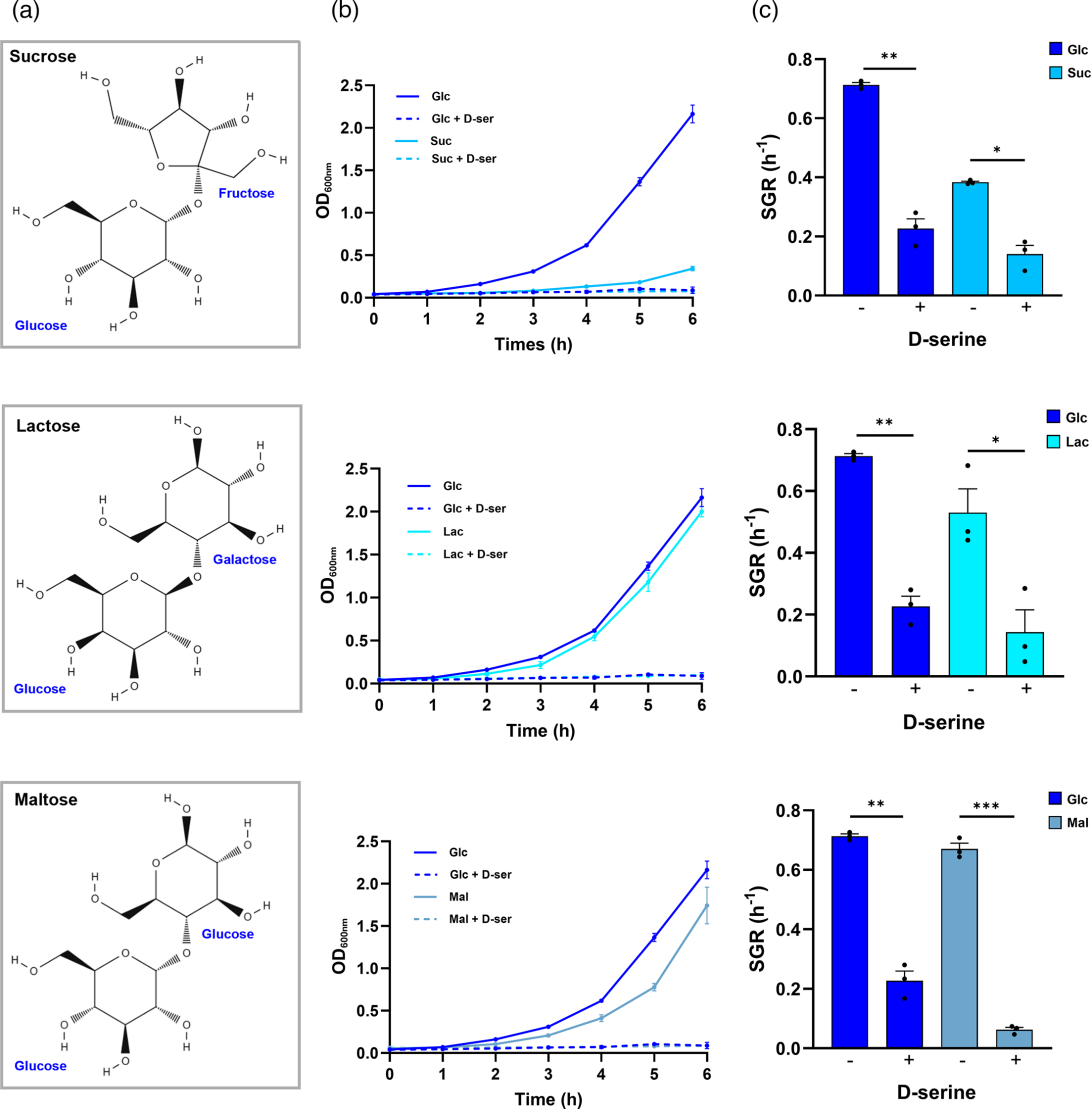
d-Serine inhibits TUV93-0 growth in M9 with alternative disaccharides. (**a**) Chemical structure of each disaccharide (black) and its constituent monosaccharides (blue). Figures adapted from [31]. (**b**) Growth curves of TUV93-0 in M9 supplemented with either glucose or an alternative disaccharide each at 0.4% (w/v), with (dashed lines) and without (solid lines) 1 mM d-serine. Error bars indicate sem. (**c**) Exponential growth rate for each disaccharide. Black dots indicate individual replicate and error bars show sem. Statistical significance: *P*<0.05 (*), *P*<0.01 (**), *P*<0.001 (***) and *P*>0.05 (ns). The data is representative of three biological replicates.

Growth was further evaluated using disaccharides sucrose, lactose and maltose as sole carbon sources (Fig. 3a). These sugars all liberate glucose during metabolism but are known to be transported by systems that function independently of SgrS. d-Serine inhibited growth across all tested disaccharides (Fig. 3b) with exponential growth rate analysis confirming statistically significant inhibition in all cases (*P*<0.05) (Fig. 3c). These findings further indicate that d-serine inhibits growth independently of sugar transport pathways regulated by SgrS.

**Fig. 4.**
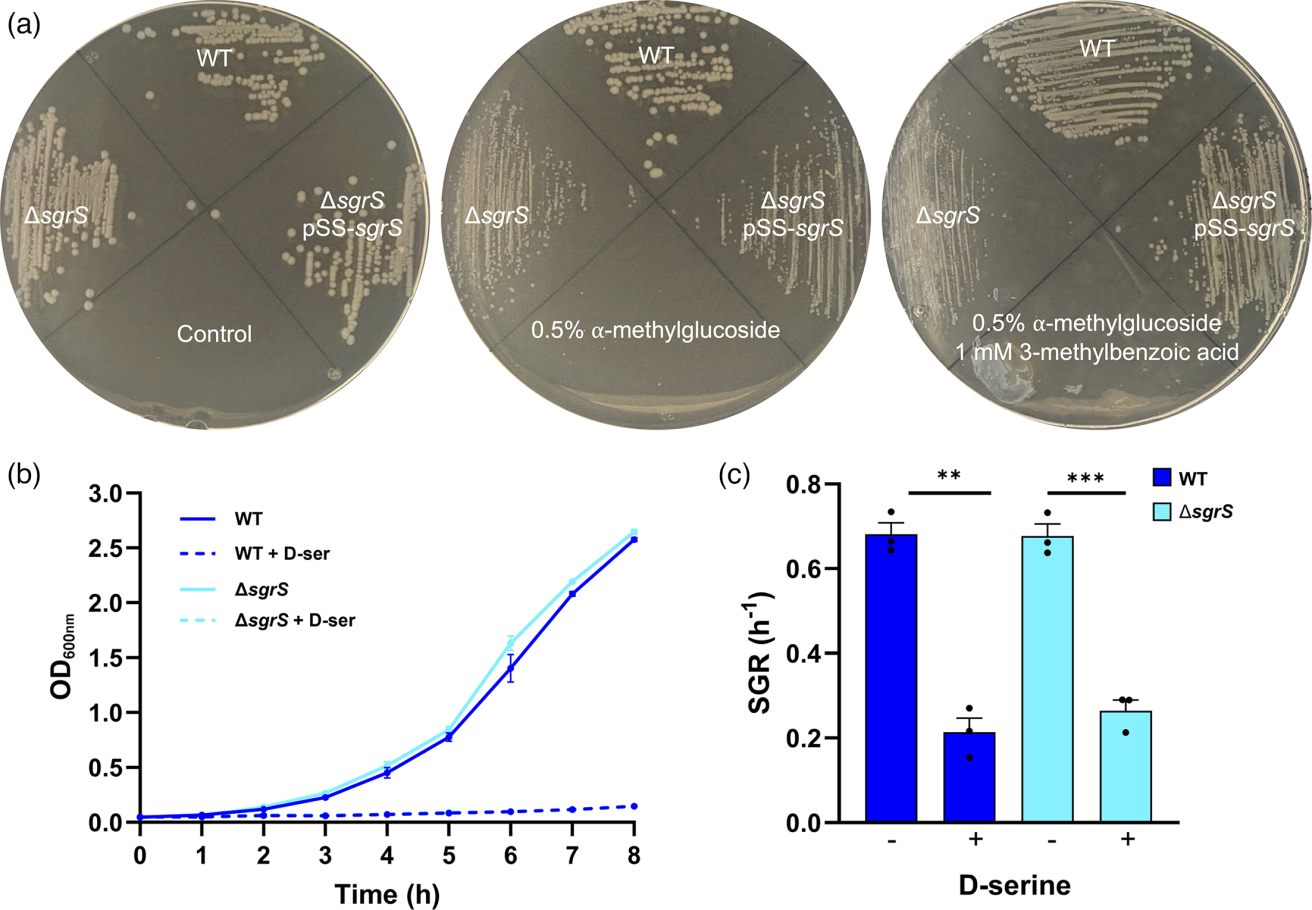
The TUV93-0 Δ*sgrS* mutant displays comparable d-serine-induced growth inhibition to the wild type (WT) in M9. (**a**) Comparison of the growth of TUV93-0 WT, Δ*sgrS* and Δ*sgrS* pSS-*sgrS* in control LB (without supplementation) and LB containing the known SgrS glucose phosphate stress-inducing metabolite *α*-MG in the presence and absence of the XylS/Pm inducer 3-methylbenzoic acid. (**b**) Growth curve of TUV93-0 WT and Δ*sgrS* in M9, with 1 mM d-serine (dashed lines) and without (solid lines). Error bars indicate sem. (**c**) Exponential growth rates for each strain. Black dots represent individual replicates. Error bars indicate sem. Significance levels are indicated as follows: *P*<0.05 (*), *P*<0.01 (**), *P*<0.001 (***) and *P*>0.05 (ns). The data was obtained from three replicate experiments.

Our sugar screening tests indicated that d-serine inhibits growth across a range of sugars that are transported independently of SgrS, suggesting that SgrS is not involved in mediating d-serine-induced growth arrest. To more directly test this, a comparison of the growth profiles of WT TUV93-0 and an isogenic Δ*sgrS* mutant in M9 glucose with and without d-serine was needed. In addition to the knock-out mutant, we constructed a complemented strain carrying *sgrS* downstream of the benzoic acid inducible Pm promoter in pSS-XylS/Pm, a synthetic construct derived from pSEVA238 [19] and pBR322 [20]. The WT, deletion mutant and complemented strain were first compared for their ability to grow in the presence of the glucose phosphate stress-inducing metabolite *⍺*-MG, a non-metabolizable synthetic analogue of glucose [15]. While all three strains grew comparably in control LB lacking any supplementation (Fig. 4a), as expected, disruption of *sgrS* resulted in a striking reduction in colony size after overnight incubation in the presence of *⍺*-MG due to its inability to tolerate the associated metabolic stress. While complementation was not apparent in the absence of inducer, inclusion of 1 mM 3-methylbenzoic acid rescued the ability of the complemented strain to grow efficiently in the presence of *⍺*-MG, demonstrating tight regulation of the XylS/Pm system and appropriate construction of the *sgrS* mutant and complemented strains (Fig. 4a).

We next compared the growth of WT TUV93-0 and our isogenic Δ*sgrS* mutant in M9 glucose. Both strains exhibited significant growth inhibition in the presence of d-serine (Fig. 4b), with comparable reductions in exponential SGRs (Fig. 4c). These data indicate that disruption of glucose uptake by SgrS does not explain the growth arrest phenotype observed in response to d-serine. Thus, SgrS is not required for the d-serine-induced inhibition of growth.

**Fig. 5.**
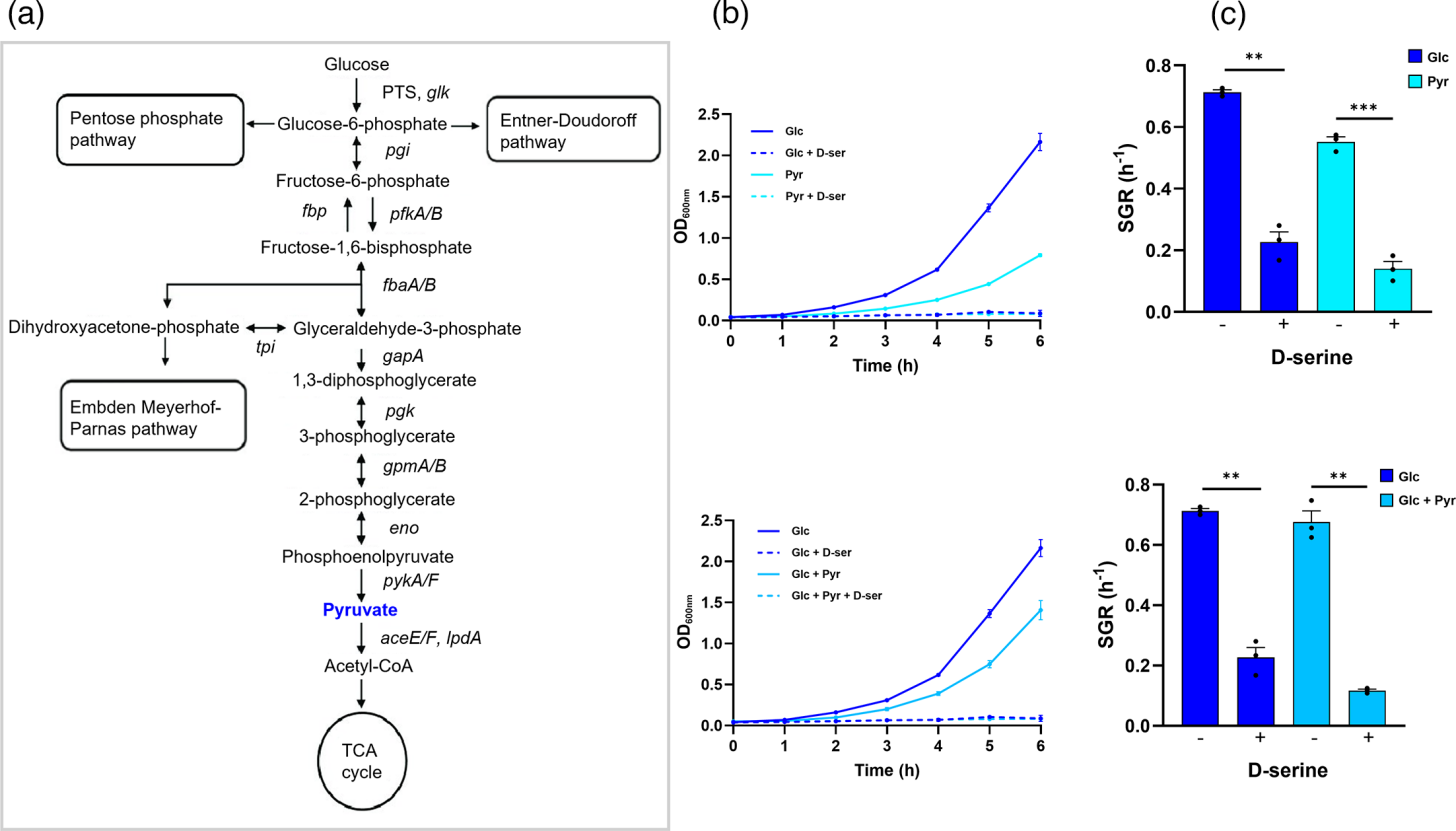
Growth of TUV93-0 in M9 medium with pyruvate is inhibited by d-serine. (**a**) Glucose metabolism in *E. coli*, figure adapted from [32,33]. (**b**) Growth curves of TUV93-0 in M9 supplemented with glucose and/or pyruvate, with (dashed lines) and without (solid lines) 1 mM d-serine. Error bars indicate sem. (**c**) Exponential growth rate for each condition. Black dots indicate individual replicate and error bars show sem. Statistical significance: *P*<0.05 (*), *P*<0.01 (**), *P*<0.001 (***) and *P*>0.05 (ns). The data is representative of three biological replicates.

Activation of SgrS has been linked to a block in glycolysis rather than directly through the accumulation of phosphosugars [22]. To assess whether d-serine toxicity results from impaired glycolysis, cultures were supplemented with exogenous pyruvate to bypass potential metabolic blockages within the glycolytic pathway. d-Serine-induced growth inhibition remained unaffected, with no improvement in M9 glucose medium even when pyruvate was the sole carbon source (Fig. 5). These observations suggest that any disruption in metabolism induced by d-serine must occur independently of glycolytic intermediate depletion.

**Fig. 6.**
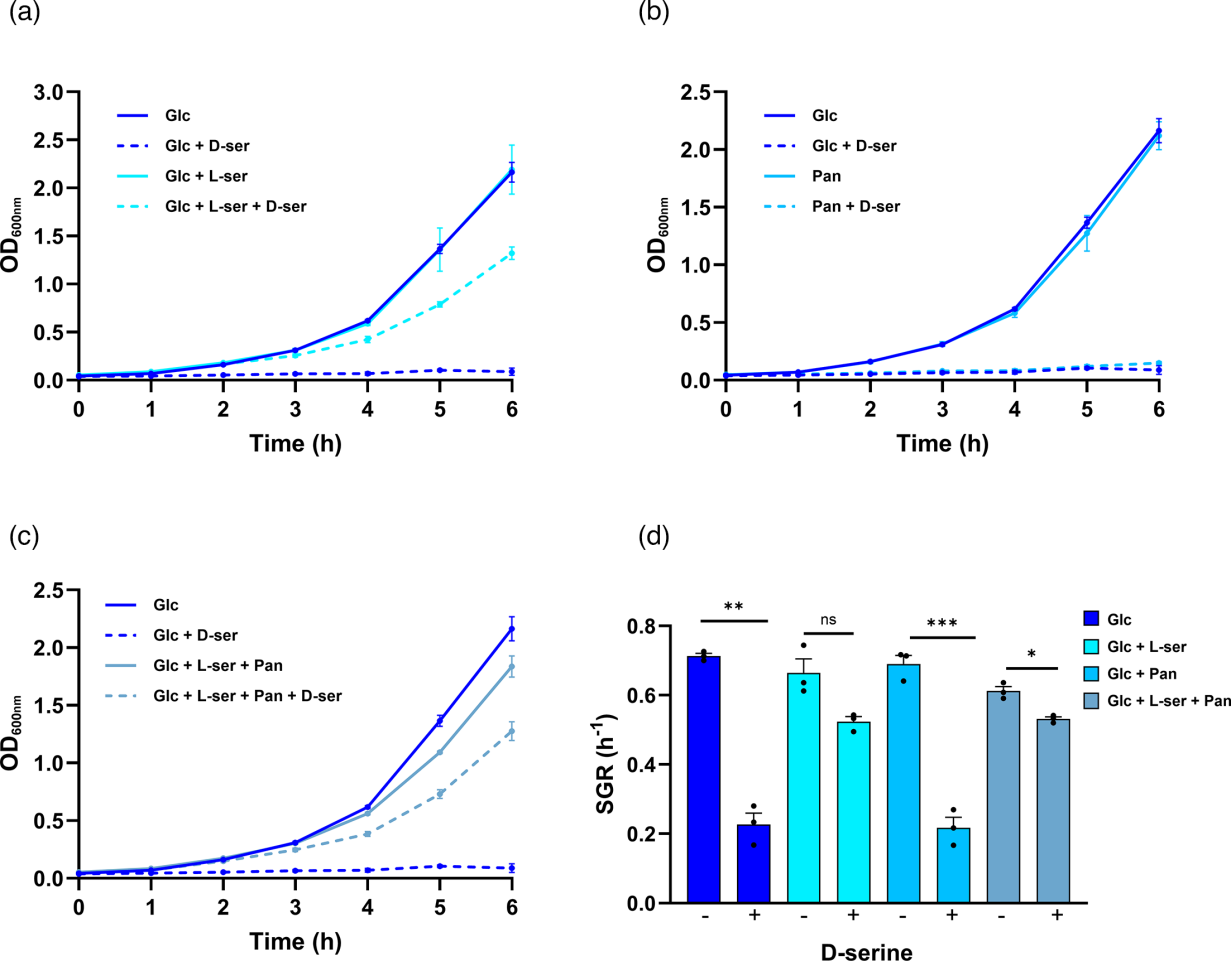
l-Serine partially rescues d-serine-induced growth arrest in TUV93-0. (**a–c**) Growth curves of TUV93-0 in M9 supplemented with glucose alone or glucose plus l-ser (**a**), calcium pantothenate (**b**) or l-ser and calcium pantothenate (**c**), with (dashed lines) and without (solid lines) d-serine. Error bars indicate sem. (**d**) Exponential growth rate for each condition. Black dots indicate individual replicates and error bars show sem. Statistical significance: *P*<0.05 (*), *P*<0.01 (**), *P*<0.001 (***) and *P*>0.05 (ns). The data is representative of three biological replicates.

To investigate potential metabolic interactions underlying d-serine toxicity, we tested the previously characterized modulators of d-serine sensitivity, l-serine and pantothenate for their ability to rescue growth. Previous work using a spontaneous *E. coli* K-12 d-serine deaminase-deficient mutant demonstrated that both l-serine and pantothenate can independently alleviate d-serine-induced growth arrest [23]. This has not been tested in WT EHEC strains which are almost exclusively naturally deficient in d-serine deaminase through loss of the transcriptional regulator DsdC [8]. Consistent with previous findings [23], l-serine partially rescued growth in TUV93-0, confirming its protective effect (Fig. 6a). However, pantothenate supplementation alone had no effect, and no additive protection was conferred when used in combination with l-serine (Fig. 6b–d). This finding suggests that d-serine toxicity in EHEC involves metabolic disruptions distinct from those seen in *E. coli* K-12.

**Fig. 7.**
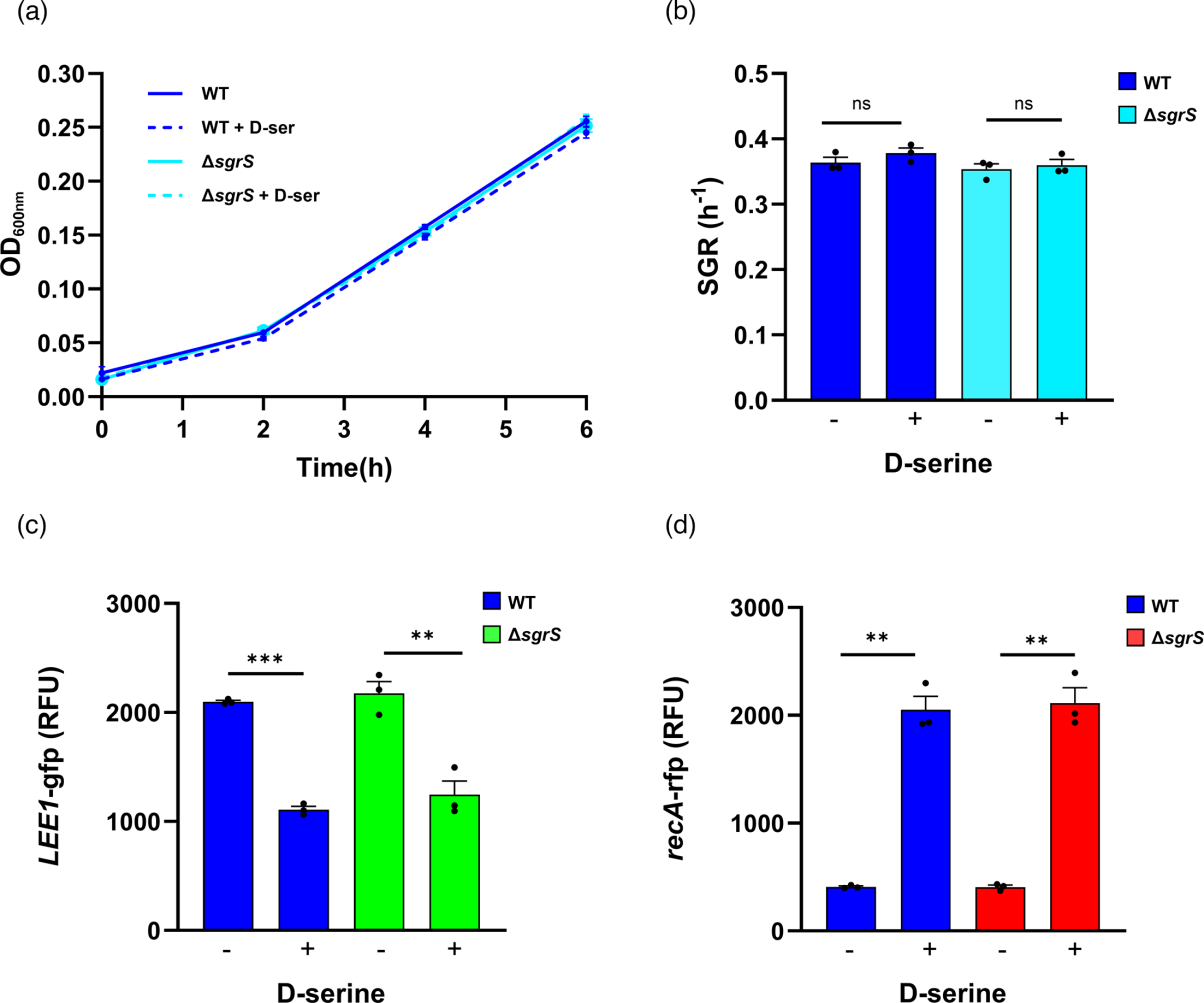
d-Serine represses *LEE1* expression and induces an SOS-like response independently of SgrS. (**a**) Growth curve of WT TUV93-0 and Δ*sgrS* in MEM, with d-serine (dashed lines) and without (solid lines). (**b**) Exponential growth rate for each strain. Black dots represent individual replicates. (**c**) *LEE1* promoter activity (GFP RFU) after 4 h in MEM±D-serine. (**d**) *recA* promoter activity (RFP RFU) after 6 h in MEM±D-serine. Black dots represent individual replicates. Error bars indicate the sem. Significance levels are indicated as follows: *P*<0.05 (*), *P*<0.01 (**), *P*<0.001 (***) and *P*>0.05 (ns). The data is representative of three replicates.

While a clear phenotype for SgrS in M9 was not determined here, we turned our attention to the known phenotypes of d-serine exposure, namely, downregulation of the LEE T3SS and upregulation of the SOS response. To test this, we employed our previously exploited dual promoter fusion reporter system [11] in MEM-HEPES, a distinct medium that is known to induce T3SS. Incidentally, d-serine does not induce EHEC growth arrest in MEM-HEPES despite activation of the SOS response [11]. Firstly, the growth of WT TUV93-0 and Δ*sgrS* was compared in MEM-HEPES, with and without d-serine supplementation. Both strains showed similar growth patterns (Fig. 7a), and as predicted, no significant growth inhibition in the presence of d-serine (Fig. 7b). In both WT and Δ*sgrS* strains, d-serine significantly repressed LEE1 expression. RecA promoter activity increased upon d-serine exposure in both strains, indicating that d-serine induces an SOS-like response and inhibits the LEE T3SS independently of SgrS (Fig. 7c, d).

## Discussion

The prediction that d-serine may act in restricting EHEC to its favoured niche within the intestine was inspired by the stark contrast in evolutionary conservation of *dsdCXA* in EHEC and UPEC isolates [8]. Further work showing downregulation of the LEE T3SS and upregulation of components of the SOS response bolstered this prediction [8,13]. While the ability of d-serine to strongly inhibit growth is medium-specific with its *in vivo* relevance yet to be shown, it is possible that this represents yet another antagonistic aspect of the effect of this host metabolite on EHEC physiology. Despite the depth of research on the interactions of d-serine with EHEC, a clear mechanism underlying its antagonistic effects has not yet been presented. This study demonstrates that d-serine-mediated growth inhibition in EHEC occurs universally across minimal media irrespective of the sole carbon source, an aspect of d-serine toxicity previously unreported. As the sugars tested each utilize distinct cellular uptake systems (Table 1), this broad-spectrum inhibition challenges our initially hypothesized link between d-serine toxicity and the SgrS-mediated glucose-phosphate stress response. This initial hypothesis was based on strong induction of SgrS in response to d-serine in glucose minimal media (Fig. 1). However, the observation that d-serine inhibits growth across diverse carbon sources, including those independent of SgrS-regulated transporters, suggests that its toxicity extends beyond inhibition of glucose uptake.

To directly determine whether SgrS mediates d-serine toxicity, growth of an isogenic Δ*sgrS* mutant was assessed. The mutant exhibited comparable growth inhibition to WT EHEC upon d-serine exposure (Fig. 4b, c), confirming that SgrS is not required for d-serine-induced growth arrest, despite its significant transcriptional activation in response to d-serine. The absence of any growth advantage in the Δ*sgrS* mutant definitively excludes SgrS-dependent glucose uptake impairment as the primary mechanism of growth inhibition. These findings indicate that SgrS upregulation reflects a secondary stress response to broader metabolic perturbations rather than a direct mechanism of growth inhibition.

Having established that d-serine inhibits growth independent of SgrS, pyruvate supplementation experiments were designed to determine the potential cause of SgrS upregulation and identify the primary mechanism of toxicity. The hypothesis here was that d-serine disrupts glycolysis, leading to an accumulation of upstream intermediates (e.g. phosphosugars) and depletion of downstream metabolites, thereby triggering SgrS upregulation as a secondary stress response. This rationale was also based on a prior study showing that supplementation of glycolytic intermediates can restore growth during glucose-phosphate stress [15]. Most carbon sources require phosphorylation before entering central metabolism, with glucose conversion to glucose-6-phosphate as the first step in glycolysis (Fig. 5a) [24]. Pyruvate, as the final glycolytic product, was selected for supplementation based on the hypothesis that it could alleviate stress caused by phosphosugar accumulation through bypassing the metabolic bottleneck. However, the failure of pyruvate to alleviate d-serine-mediated growth inhibition provides compelling evidence against simple glycolytic disruption as the primary mechanism of toxicity (Fig. 5b, c). In addition, deletion of *sgrS* was not associated with augmentation of the growth inhibitory effect of d-serine, excluding SgrS induction as a productive means of mitigating d-serine stress (Fig. 4b, c). Instead, d-serine may target metabolic processes downstream of pyruvate, such as the tricarboxylic acid (TCA) cycle, which could indirectly lead to phosphosugar accumulation and thus trigger the upregulation of SgrS. This finding prompted further investigation into alternative metabolic interventions to identify the specific cellular processes most affected by d-serine toxicity.

Pantothenate was investigated as a potential metabolic rescue agent based on a previous study in a spontaneous *E. coli* K-12 d-serine deaminase-deficient mutant where supplementation successfully alleviated d-serine toxicity [23]. It is suggested that d-serine interferes with pantothenate synthesis, a precursor for coenzyme A (CoA) formation [23]. This could impact growth by impeding fatty acid metabolism or disrupting the TCA cycle. Further support for investigating pantothenate arose from differences in d-serine toxicity across growth media. EHEC does not exhibit growth inhibition in the presence of d-serine in MEM-HEPES medium (Fig. 7a), which contains pantothenate, whereas significant inhibition occurs in M9 minimal medium, which lacks pantothenate [8]. As CoA is an essential cofactor for the delivery of acetate to the TCA cycle downstream of pyruvate, pantothenate supplementation represented a logical extension of the metabolic rescue strategy and could theoretically also prevent the accumulation of upstream metabolites, such as phosphosugars. Rescue experiments therefore used 1 µg ml^−1^ calcium pantothenate, matching the level in standard MEM-HEPES and 100-fold higher than the concentration (0.01 µg ml^−1^) used by Cosloy and McFall [23]. However, pantothenate failed to restore growth of TUV93-0 in d-serine-supplemented M9 minimal medium (Fig. 6b), indicating that d-serine toxicity in EHEC may operate through mechanisms distinct from those previously observed in K-12 d-serine deaminase mutants. The persistent growth arrest in TUV93-0 suggests fundamental metabolic differences between clinical and laboratory *E. coli* isolates and points to either alternative, potentially strain-specific cellular targets for d-serine beyond CoA biosynthesis or several simultaneous inhibitory mechanisms that cannot be resolved by pantothenate alone.

The study by Cosloy and McFall also demonstrated that l-serine supplementation can counteract d-serine toxicity in the same d-serine deaminase mutant [23]. Following unsuccessful rescue attempts with pyruvate and pantothenate, l-serine was tested. At an equimolar concentration to d-serine, l-serine significantly alleviated d-serine-mediated growth inhibition (Fig. 6a). The primary explanation for this rescue effect presented by Cosloy and McFall is that d-serine interferes with l-serine biosynthesis, likely inhibiting SerA or SerC (phosphoglycerate dehydrogenase and phosphoserine aminotransferase), which respectively convert phosphoglycerate to phosphohydroxypyruvate and phosphohydroxypyruvate to phosphoserine [23]. Inhibition of either enzyme by d-serine may cause phosphoglycerate or phosphohydroxypyruvate (phosphorylated intermediates) to accumulate, potentially eliciting a stress response analogous to glucose-phosphate stress and thereby driving SgrS upregulation. Supplementing with exogenous l-serine would bypass this block and alleviate toxicity. Beyond l-serine biosynthetic inhibition, d-serine may disrupt nitrogen metabolism by competing with l-serine in transamination reactions due to their structural similarity [25]. As transaminase enzymes rely on substrate recognition, d-serine could interfere with amino acid biosynthesis and nitrogen distribution, leading to functional nitrogen limitation despite sufficient environmental nitrogen. Indeed, the nitrogen stress response components encoded by *glnALG* are amongst the most strongly induced (2.94-, 4.25- and 3.34-fold respectively, with false discovery rate-adjusted *P*=1.18×10^−7^, 6.78×10^−9^ and 1.25×10^−5^) d-serine responsive genes in EHEC [14]. This nitrogen competition hypothesis suggests that future experiments testing l-serine as a sole nitrogen source could provide further mechanistic insights. Additionally, investigating competition dynamics between d-serine and l-amino acids present in MEM-HEPES may further clarify broader metabolic interactions involved in d-serine toxicity.

Beyond its growth effects, d-serine represses the LEE-encoded T3SS in EHEC, establishing a link between metabolism and virulence [26]. While no direct base pairing of SgrS with T3SS transcripts was predicted, a contribution to virulence remained plausible given the role of SgrS in regulating carbon source uptake and our recently published report on PdhR, a T3SS-inducing transcription factor which responds to endogenous pyruvate [27]. To investigate SgrS contribution to T3SS regulation, reporter assays measuring *LEE1* activity were performed. No significant difference in *LEE1* expression was observed between WT and Δ*sgrS* strains with and without d-serine treatment, indicating that SgrS is not a fundamental regulator of the T3SS under these conditions and is not required for repression of the LEE in response to d-serine (Fig. 7c). This finding aligns with the initial expectation, as SgrS is not known to directly regulate LEE-associated mRNAs. However, given the post-transcriptional regulatory function of SgrS, its influence may be more apparent at the level of protein secretion or host interaction. Future experiments could explore potential roles in effector translocation or epithelial cell infection models, where post-transcriptional effects might be more easily detected.

d-Serine also induces *recA* expression in EHEC, activating an SOS-like response, likely a strategy to overcome the stress of d-serine [13]. Given the observation that SgrS is upregulated during d-serine exposure and functions in stress regulation, it was important to determine whether it contributes to *recA* activation. Reporter assays showed equivalent *recA* activation in WT and Δ*sgrS*, confirming that SgrS is not required for SOS induction (Fig. 7c). *RecA* activation therefore occurs via an SgrS-independent route, demonstrating d-serine’s capacity to mobilize multiple stress pathways in parallel.

This study progresses our understanding of d-serine toxicity in *E. coli* O157:H7 in several ways. Firstly, the finding that SgrS is induced by d-serine is novel and suggests a disruption in carbon metabolism. Secondly, as pantothenate was unable to restore growth in EHEC, there is a clear distinction between d-serine toxicity in EHEC and laboratory *E. coli* strains. Thirdly, as neither deletion of *sgrS* nor supplementation with pyruvate overcame d-serine toxicity, we can definitively exclude the canonical glucose phosphate stress response mediated by SgrS as the primary driver of growth inhibition. Finally, we show that in agreement with previous studies using *E. coli* K-12 derived mutants, l-serine partially restores the growth of EHEC in d-serine. Dissecting the precise mechanism underlying this is not trivial. Interference with serine biosynthesis through inhibition of key enzymes such as SerA or SerC, competition in transamination reactions and disruption of nitrogen assimilation are all possible explanations. Beyond its canonical role in the regulation of glucose uptake, SgrS also represses *asd*, *adiY*, *folE* and *purR*, which are involved in amino acid, purine and folate metabolism [28] and may therefore contribute to broader cellular responses. Together, these results identify SgrS as a sensitive marker of metabolic stress including stress associated with d-serine. This study adds to a growing body of research highlighting the complex antagonistic effects of d-serine on *E. coli* strains that lack the ability to detoxify this host-produced metabolite. This work reinforces the interconnected nature of metabolism, stress and virulence regulation in bacterial pathogens.

## Supplementary material

10.1099/mic.0.001648 Uncited Supplementary Material 1.

## Abbreviations

EHEC: enterohaemorrhagic *Escherichia coli*
HUS: haemolytic uraemic syndrome
LB: lysogeny broth
LEE: locus of enterocyte effacement
PTS: phosphotransferase system
RFU: relative fluorescence units
SGR: specific growth rate
SOC: super optimal complete
TCA: tricarboxylic acid
T3SS: type III secretion system
UPEC: uropathogenic *E.coli*
*α*-MG: *α*-methylglucoside

## References

[1] Martinson JNV, Walk ST (2020). *Escherichia coli* residency in the gut of healthy human adults. EcoSal Plus.

[2] Wang F, Sun H, Kang C, Yan J, Chen J (2024). Genomic island-encoded regulatory proteins in enterohemorrhagic *Escherichia coli* O157:H7. Virulence.

[3] Joseph A, Cointe A, Mariani Kurkdjian P, Rafat C, Hertig A (2020). Shiga toxin-associated hemolytic uremic syndrome: a narrative review. Toxins.

[4] Lange ME, Uwiera RRE, Inglis GD (2022). Enteric *Escherichia coli* O157:H7 in cattle, and the use of mice as a model to elucidate key aspects of the host-pathogen-microbiota interaction: a review. Front Vet Sci.

[5] Stevens MP, Frankel GM (2014). The locus of enterocyte effacement and associated virulence factors of enterohemorrhagic *Escherichia coli*. Microbiol Spectr.

[6] Sy BM, Lan R, Tree JJ (2020). Early termination of the Shiga toxin transcript generates a regulatory small RNA. Proc Natl Acad Sci USA.

[7] Feitz WJC, Bouwmeester R, van der Velden TJAM, Goorden S, Licht C (2021). The Shiga toxin receptor globotriaosylceramide as therapeutic target in Shiga toxin *E. coli* mediated HUS. Microorganisms.

[8] Connolly JPR, Goldstone RJ, Burgess K, Cogdell RJ, Beatson SA (2015). The host metabolite D-serine contributes to bacterial niche specificity through gene selection. ISME J.

[9] Puño-Sarmiento J, Anderson EM, Park AJ, Khursigara CM, Barnett Foster DE (2020). Potentiation of antibiotics by a novel antimicrobial peptide against Shiga toxin producing *E. coli* O157:H7. Sci Rep.

[10] Mustafa AK, Kim PM, Snyder SH (2004). D-Serine as a putative glial neurotransmitter. Neuron Glia Biol.

[11] O’Boyle N, Connolly JPR, Tucker NP, Roe AJ (2020). Genomic plasticity of pathogenic *Escherichia coli* mediates D-serine tolerance via multiple adaptive mechanisms. Proc Natl Acad Sci USA.

[12] Parveen S, Reddy M (2017). Identification of YfiH (PgeF) as a factor contributing to the maintenance of bacterial peptidoglycan composition. Mol Microbiol.

[13] Connolly JPR, Roe AJ (2016). Intracellular D-serine accumulation promotes genetic diversity via modulated induction of RecA in enterohemorrhagic *Escherichia coli*. J Bacteriol.

[14] Connolly JPR, Turner NCA, Hallam JC, Rimbi PT, Flett T (2021). D-serine induces distinct transcriptomes in diverse *Escherichia coli* pathotypes. Microbiology.

[15] Richards GR, Patel MV, Lloyd CR, Vanderpool CK (2013). Depletion of glycolytic intermediates plays a key role in glucose-phosphate stress in *Escherichia coli*. J Bacteriol.

[16] Raina M, Storz G (2017). SgrT, a small protein that packs a sweet punch. J Bacteriol.

[17] Wadler CS, Vanderpool CK (2007). A dual function for a bacterial small RNA: SgrS performs base pairing-dependent regulation and encodes a functional polypeptide. Proc Natl Acad Sci USA.

[18] Horler RSP, Vanderpool CK (2009). Homologs of the small RNA SgrS are broadly distributed in enteric bacteria but have diverged in size and sequence. Nucleic Acids Res.

[19] Calles B, Goñi-Moreno Á, de Lorenzo V (2019). Digitalizing heterologous gene expression in Gram-negative bacteria with a portable ON/OFF module. Mol Syst Biol.

[20] Balbás P, Soberón X, Merino E, Zurita M, Lomeli H (1986). Plasmid vector pBR322 and its special-purpose derivatives--a review. Gene.

[21] Springthorpe V, Leaman R, Sifouna D, Bennett J, Thomas G (2020). MORF: an online tool for exploring microbial cell responses using multi-omics analysis. Access Microbiol.

[22] Boulanger EF, Sabag-Daigle A, Thirugnanasambantham P, Gopalan V, Ahmer BMM (2021). Sugar-phosphate toxicities. Microbiol Mol Biol Rev.

[23] Cosloy SD, McFall E (1973). Metabolism of D-serine in *Escherichia coli* K-12: mechanism of growth inhibition. J Bacteriol.

[24] Chowdhury S, Hepper S, Lodi MK, Saier MH Jr, Uetz P (2021). The protein interactome of glycolysis in *Escherichia coli*. Proteomes.

[25] Okuda J, Nagata S, Yasuda M, Suezawa C (2019). Validating the inhibitory effects of d- and l-serine on the enzyme activity of d-3-phosphoglycerate dehydrogenases that are purified from *Pseudomonas aeruginosa*, *Escherichia coli* and human colon. Gut Pathog.

[26] Connolly JPR, Gabrielsen M, Goldstone RJ, Grinter R, Wang D (2016). A highly conserved bacterial D-serine uptake system links host metabolism and virulence. PLoS Pathog.

[27] Wale KR, O’Boyle N, McHugh RE, Serrano E, Mark DR (2024). A master regulator of central carbon metabolism directly activates virulence gene expression in attaching and effacing pathogens. PLoS Pathog.

[28] Bobrovskyy M, Vanderpool CK (2016). Diverse mechanisms of post-transcriptional repression by the small RNA regulator of glucose-phosphate stress. Mol Microbiol.

[29] Keseler IM, Gama-Castro S, Mackie A, Billington R, Bonavides-Martínez C (2021). The EcoCyc database in 2021. Front Microbiol.

[30] Stephens C, Martinez M, Leonardi V, Jaing J, Miller A (2024). The Scr and Csc pathways for sucrose utilization co-exist in *E. coli*, but only the Scr pathway is widespread in other *Enterobacteriaceae*. Front Microbiol.

[31] Smith TJ (1995). MolView: a program for analyzing and displaying atomic structures on the Macintosh personal computer. J Mol Graph.

[32] Phong WY, Lin W, Rao SPS, Dick T, Alonso S (2013). Characterization of phosphofructokinase activity in *Mycobacterium tuberculosis* reveals that a functional glycolytic carbon flow is necessary to limit the accumulation of toxic metabolic intermediates under hypoxia. PLoS One.

[33] Martínez-Gómez K, Flores N, Castañeda HM, Martínez-Batallar G, Hernández-Chávez G (2012). New insights into *Escherichia coli* metabolism: carbon scavenging, acetate metabolism and carbon recycling responses during growth on glycerol. Microb Cell Fact.

