# Supplementary material for: The glucose uptake inhibitor SgrS is induced by D-serine yet does not contribute to growth arrest in enterohaemorrhagic Escherichia coli
Source: Microbiology (Reading). 2026 Jan 9;172(1):001648. doi: 10.1099/mic.0.001648 (PMC13293527; doi:10.1099/mic.0.001648)
Supplement: Uncited Supplementary Material 1. [file mic-172-01648-s001.pdf]

**Supplementary Information**

# The glucose uptake inhibitor SgrS is induced by D-serine yet does not contribute to growth arrest in enterohaemorrhagic *E. coli*

Ella Rellis and Nicky O'Boyle

**Table S1. Bacterial strains**

| Strain | Description | Reference |
|---|---|---|
| EHEC TUV93-0 | Wild type *E. coli* O157:H7 (Stx negative) | (1) |
| EHEC Δ*sgrS* | TUV93-0 mutant lacking *sgrS*; kanR | This study |
| Δ*sgrS* pSS-*sgrS* | TUV93-0 mutant lacking *sgrS* carrying *sgrS* on pSS expression plasmid; kanR; ampR | This study |
| DH5α | Chemically competent *E. coli* | (2) |
| TUV pSIM18 | WT carrying pSIM18 plasmid; hygR | O'Boyle Group Inventory |
| TUV pDUAL | WT carrying pDUAL plasmid; chlR | O'Boyle Group Inventory |
| TUV pLEE1/RecA | WT carrying pLEE1/RecA plasmid; chlR | O'Boyle Group Inventory |
| Δ*sgrS* pDUAL | TUV93-0 mutant; pDUAL; kanR; chlR | This study |
| Δ*sgrS* pLEE1/RecA | TUV93-0 mutant; pLEE1/RecA; kanR; chlR | This study |

kanR: kanamycin resistance; ampR: ampicillin resistance; chlR: chloramphenicol resistance; hygR: hygromycin B resistance.

**Table S2. Plasmids**

| Plasmid | Description | Reference |
|---|---|---|
| pKD4 | Lambda red template; DH5α lambda pir; kanR | (3) |
| pACYC184 | Shuttle vector with p15A origin of replication; chlR | (4) |
| pSIM18 | Lambda red helper plasmid; 28°C temperature sensitive origin; 42°C inducible red recombinase system; hygR | (5) |
| pSS-XylS/Pm (pSS) | Synthetic construct derived from pBR322 and pSEVA238 for benzoic acid inducible expression; ampR | This study |
| pSS-*sgrS* | pSS-XylS/Pm with *sgrS* cloned downstream of Pm promoter for benzoic acid inducible expression; ampR | This study |
| pDUAL | Dual expression vector; empty backbone; chlR | (6) |
| pLEE1-gfp+recA-rfp | Expression plasmid containing *LEE1* promoter driving GFP and *recA* promoter driving RFP; chlR | (6) |

kanR: kanamycin resistance; chlR: chloramphenicol resistance; ampR: ampicillin resistance; hygR: hygromycin B resistance.

**Table S3. Oligonucleotides**

| Oligo | Sequence | Description |
|---|---|---|
| sgrS-DelA | gcacctgaagtcagccccatacgatTCACGTCGTCCATTTCCAG | *sgrS* deletion |
| sgrS-DelB | acttcgaagcagctccagcctacacCTGTCTTTTGCGCAGGAG | *sgrS* deletion |
| sgrS-DelC | aggaactaaggaggatattcatatgCGGGTTGCTTCGCTTCATAAG | *sgrS* deletion |
| sgrS-DelD | gcctacaatccatgccaacccgttcATAAGGAGCACACCATGG | *sgrS* deletion |
| sgrS-check-F | GATCATCGCCGGAACTTGTC | *sgrS* deletion check |
| sgrS-check-R | TGGCTGCTCAATGTGTCTG | *sgrS* deletion check |
| pACYC-lin-F | GAACGGGTTGGCATGGATTG | pACYC linearisation |
| pACYC-lin-R | ATCGTATGGGGCTGACTTCA | pACYC linearisation |
| pKD-F | GTGTAGGCTGGAGCTGCTTC | Kan cassette amplification |
| pKD-R | CATATGAATATCCTCCTTAG | Kan cassette amplification |
| pACYC-seq2-F | CAAGAGATTACGCGCAGACC | Assembly check |
| pACYC-check-R | GCATTCACAGTTCTCCGCAA | Assembly check |
| pACYC-seq3-F | AGCCAGTTACCTCGGTTCAA | Sequencing |
| pSS-lin-F | AACCGGGGATCCTCTAG | pSS linearisation |
| pSS-lin-R | TACCGAGCTTTCTCCTCTTTAATTC | pSS linearisation |
| sgrS-pSS F | aaagaggagaaagctcggtaGATGAAGCAAGGAGGTGC | *sgrS* complementation |
| sgrS-pSS R | ctctagaggatccccgggttTTATGAAGCGAAGCAACC | *sgrS* complementation |
| pSS-check-F | GCAGTGTCCGGTTTGATAGG | Assembly check |
| pSS-check-R2 | AACCGAGCGTTCTGAACAAA | Assembly check |

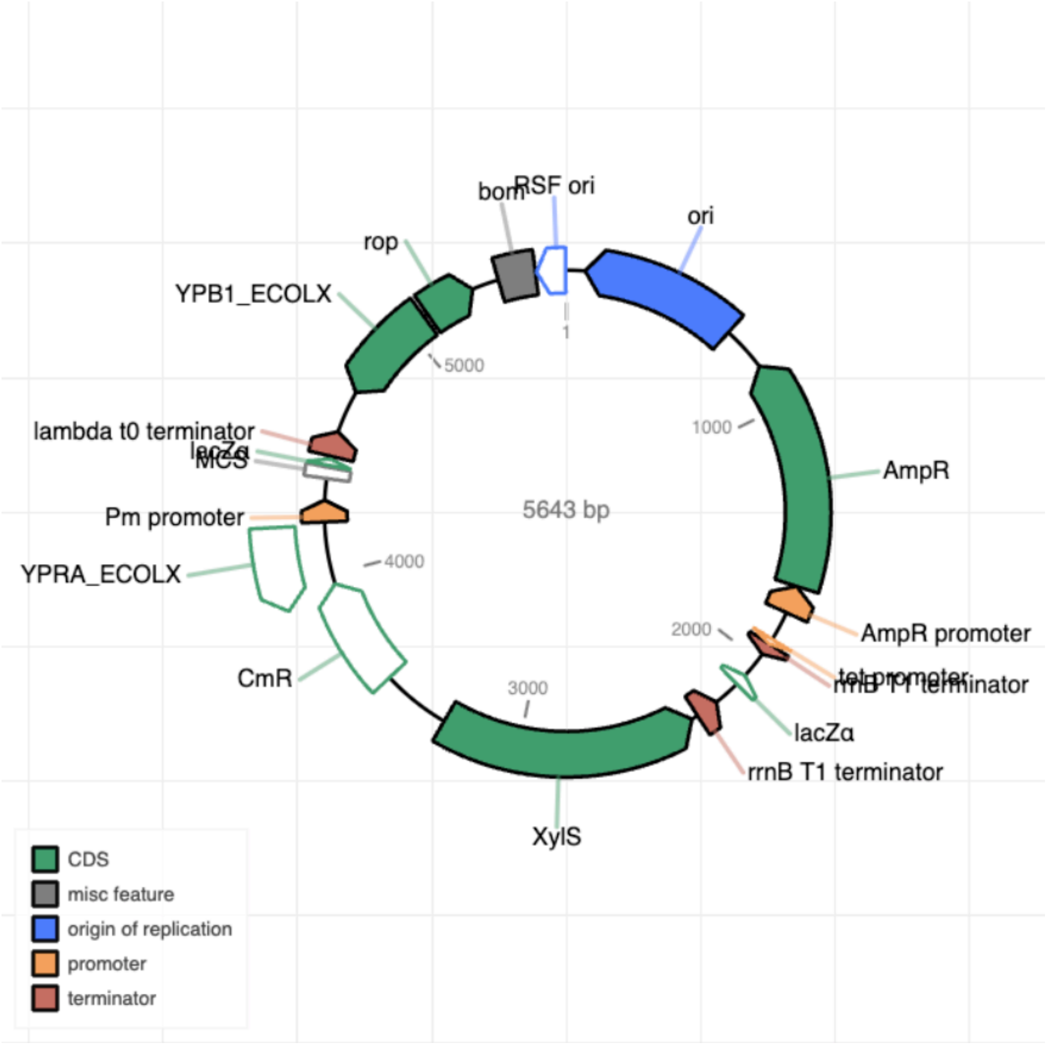


**Fig. S1 Map of synthetic pSS-XylS/Pm plasmid.** Map was generated using pLANNOTATE with default settings (7). The sequence of this construct has been uploaded to NCBI (Accession: PX119074).

**Supplementary Information References**

1. **Campellone KG, Giese N, Tipper OJ, Leong JM.** A tyrosine-phosphorylated 12-amino-acid sequence of enteropathogenic *Escherichia coli* Tir binds the host adaptor protein Nck and is required for Nck localization to actin pedestals. Molecular Microbiology. 2002;43(5):1227-41.
2. **Hanahan D.** Studies on transformation of *Escherichia coli* with plasmids. Journal of Molecular Biology. 1983;166(4):557-80.
3. **Datsenko KA, Wanner BL.** One-step inactivation of chromosomal genes in *Escherichia coli* K-12 using PCR products. Proceedings of the National Academy of Sciences. 2000;97(12):6640-5.
4. **Chang AC, Cohen SN.** Construction and characterization of amplifiable multicopy DNA cloning vehicles derived from the P15A cryptic miniplasmid. Journal of Bacteriology. 1978;134(3):1141-56.
5. **Chan W, Costantino N, Li R, Lee SC, Su Q, Melvin D, et al.** A recombineering based approach for high-throughput conditional knockout targeting vector construction. Nucleic Acids Research. 2007;35(8):e64-e.
6. **O'Boyle N, Connolly JPR, Tucker NP, Roe AJ.** Genomic plasticity of pathogenic *Escherichia coli* mediates D-serine tolerance via multiple adaptive mechanisms. Proceedings of the National Academy of Sciences. 2020;117(36):22484-93.
7. **McGuffie MJ, Barrick JE.** pLannotate: engineered plasmid annotation. Nucleic Acids Research. 2021;49(W1):W516-W22.
